# Using retrospective life tables to assess the effect of extreme climatic conditions on ungulate demography

**DOI:** 10.1002/ece3.8218

**Published:** 2021-12-24

**Authors:** Marta Peláez, Alfonso San Miguel, Carlos Rodríguez‐Vigal, Ángel Moreno‐Gómez, Amanda García del Rincón, Ramón Perea García‐Calvo

**Affiliations:** ^1^ Departamento de Sistemas y Recursos Naturales Universidad Politécnica de Madrid Madrid Spain; ^2^ Centro de Quintos de Mora Toledo Spain; ^3^ Organismo Autónomo Parques Nacionales Madrid Spain

**Keywords:** cervid, drought, dynamic, global warming, life table, population, ungulates

## Abstract

In Mediterranean areas, severe drought events are expected to intensify in forthcoming years as a consequence of climate change. These events may increase physiological and reproductive stress of wild populations producing demographic changes and distribution shifts. We used retrospective life tables to understand demographic changes on a wild population after severe drought events. We studied the impact of two extreme events (2003 and 2005) on the population dynamics of our model species, the red deer (*Cervus elaphus*). During both years, population density was high (40 and 36 ind/100 ha, respectively). Thus, we reconstructed retrospectively the age structure of the female part of the population for the period 2000–2010 by using data of known‐age individuals culled during the period 2000–2019 (*n* = 4176). Also, based on previous study results, we aimed to validate this methodology. Both extremely dry years, 2003 and 2005, produced marked and lasting cohort effects on population demography. Age pyramid the following years (2004 and 2006) revealed that the extreme drought caused the female fawn cohort to be similar or even smaller than the yearling cohort. Furthermore, these cohort effects were still perceptible 3 years after these severe events. Results agree with previous findings that showed a negative effect of severe drought events on female pregnancy rates and conception dates. Although simple, this study provides an empirical quantification of the demographic effects of severe drought events for a wild population which might be useful to understand future demographic changes under the context of climate change.

## Graphical abstract

Retrospective life tables for wild populations of animals might be a useful tool to assess future demographic changes under the context of climate change. Using this methodology, we provided an empirical quantification of the demographic effects of severe drought events for a wild red deer population.

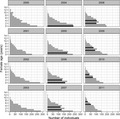

## 1 INTRODUCTION

Populations living at the edge of a species’ range often experience extreme conditions and different selective pressures than their analogs living in more central locations of their geographic distribution (Kawecki, 2008). Therefore, long‐term studies performed on these regions may provide a clue about how populations may respond to future climatic scenarios, helping to predict demographic changes and distribution shifts (Büntgen et al., 2017; Chevin & Hoffmann, 2017; Plard et al., 2014).

Retrospective life tables (i.e., age pyramids) for wild populations might be a useful tool to better understand demographic changes after extreme events, which may be interesting under the current context of climate change. This technique was first proposed by Fry (1949) to analyze minimum population size of lake trout (*Salvelinus namayacush*) in North America. This method allowed the retrospective estimation of the minimum number of individuals alive for each trout cohort and year using harvest data from subsequent years, and thus allowing the estimation of several demographic parameters through the construction of age pyramids. However, the following three main assumptions must be met: (a) Age can be determined with accuracy; (b) there is no migration of individuals into, or out of, the population as the technique assumes that if an individual is hunted at age *t*, it was present in the area during the previous *t* years; and (c) sufficient time is given after each cohort can be calculated. Therefore, this method can be used for terrestrial animals such as ungulates in fenced areas or otherwise isolated populations (such as those living in islands) as they function as closed environment similarly to lakes (see Lowe, 1969 on red deer; McCulloug, 1979 on white‐tailed deer *Odocoileus virginianus*; Fryxell et al., 1988 on moose).

Retrospective life tables may be useful to quantify the demographic effect produced by extreme drought events and thus to quantify the possible consequences of climate change on wild ungulate populations. However, this technique has seldom been used in recent times, even though there is a lot of detailed hunting data available across the Northern Hemisphere. The challenges of maintaining funding and consistent data collection throughout decades (Festa‐Bianchet et al., 2017) may be one of the main drawbacks of this technique.

Climate changes due to global warming are predicted to be especially marked in the Mediterranean region where climate models predict an increase in the frequency and severity of droughts in the forthcoming years (Hoerling et al., 2012; IPCC, 2013; Peñuelas et al., 2002). This could impose a further constraint to the reproductive cycle of many species, particularly for those species for which the Mediterranean environment constitutes the southern limit of their distribution. Indeed, this is the case of the red deer (*Cervus elaphus* L.), our model species (Lovari et al., 2018). For red deer females living in Mediterranean environments, lactation (the period of highest energy requirements) overlaps with the summer drought (the period of highest nutritional constraint when most pastures are withered). This can be energetically draining for females which, some years, may have to endure 3–5 months of severe summer drought (from May to September; Bugahlo & Milne, 2003; San Miguel et al., 1999).

The effects of a mismatch between parturition date or lactation and resource availability on cervids can have long‐lasting demographic consequences for the population (Plard et al. (2014) on roe deer), as it decreases both calf survival and female reproductive success the following season. For most cervid species, parturition date is mainly determined by conception date (Clements et al. (2011) but see Short & Hay (1966) on roe deer). Indeed, for red deer, delayed ovulation is usually the result of decreased female condition as it has been frequently reported in high‐density populations. Furthermore, in Mediterranean environments, after a dry year (i.e., short spring followed by a long summer drought period), lactating females deplete their body fat reserves which leads to lower pregnancy rates (Rodríguez‐Hidalgo et al., 2010) or delayed and less synchronous conception dates (Pelaez et al., 2017). These late conception dates after a dry year could create a potential mismatch between birth date and plant phenology the following year, which could jeopardize calf survival (Clutton‐Brock, Iason, et al., 1987; Clutton‐Brock, Major, et al., 1987; Plard et al., 2014) and hind reproductive success the following season (Clutton‐Brock et al., 1983). If gestation length cannot compensate the delay on parturition date (Clements et al., 2011; Scott et al., 2008), it is expected that climate change in Mediterranean environments could produce a negative impact on red deer demography.

In other areas such as the temperate zone, red deer response to climate change seems to be the opposite as that reported for Mediterranean populations. Previous findings on the Island of Rhum, Scotland, described that red deer advanced breeding phenology as a response to climate change (Moyes et al., 2011). In these environments, the advancement of spring plant phenology decreased the length and severity of winter (the limiting season) and increased spring–summer resource availability. Hence, red deer females increased body condition throughout the summer, conceiving sooner in the season and thus, giving birth earlier through time, keeping in synchrony with the earlier spring phenology, although no clear effect on demography was found (Moyes et al., 2011). However, in Mediterranean environments, a delay in conception timing was observed with increasing dry conditions (Pelaez et al., 2017). Thus, the plastic phenotypic response observed on red deer conception dates in these environments may produce a mismatch between resource availability and energy need producing a cohort effect (i.e., reduction of the fawn cohort) after a severe drought event. Furthermore, this marked reduction in recruitment, if maintained thought time, might have a long‐lasting effect in the population structure and thus on its demography as the variation in recruitment is known to be the main driver of observed demographic change for other deer species in water‐stressed environments (see Gaillard et al., 2013 on roe deer).

Here, we studied a red deer population living in a Mediterranean environment located in Toledo (Central Spain). In this fenced area, a high percentage of the deer population is harvested (~20%) each year in order to reduce population density as it was very high at the beginning of the study and there are no natural predators. For all individuals culled or found dead, the age of individuals was accurately assessed by using cementum annulus counts (Mitchell, 1967).

We hypothesize that:

H1: After an extreme drought event, fawn cohort size (expressed as the percentage of fawns in the age pyramid) will be significantly reduced compared with the previous year cohort size (percentage of yearlings in the same age pyramid).
H2: Age pyramid results will match previous findings on the same population. These studies signaled toward lower pregnancy rates (Rodríguez‐Hidalgo et al., 2010) and later conception dates after severe drought events with high density (Pelaez et al., 2017). This would in turn provoke (a) less females giving birth but also (b) a higher mortality near birth and lower summer survival as a consequence of a trophic mismatch due to a delayed birth date. Thus, we expect these consequences to be reflected in the age pyramids.

## 2 MATERIAL AND METHODS

### 2.1 Study area

Data were collected from Los Quintos de Mora, a perimeter‐fenced hunting estate of 6864 hectares located in a hilly area in southcentral Spain (39°26′N, 4°2′W). The climate is Continental Mediterranean, characterized by 3–5 months of dry, hot summer and high inter‐annual variability of rainfall. The estate is dominated by Mediterranean vegetation, and the most common tree species is Holm oak (*Quercus ilex*), which coexists with a variety of evergreen shrubs such as those belonging to the genera *Cistus*, *Rosmarinus*, *Rubus*, *Erica*, *Phillyrea*, and *Cytisus*. In the lower areas, there are scattered woody grasslands “Dehesas” and croplands [more details on the study area in Peláez et al. (2017)].

The main game species is red deer, which move freely inside the fenced estate and feed mainly on natural vegetation. However, they have access to 2.1 km^2^ of rainfed crops (oats, barley, rye, common vetch, and clover) which are protected by an electric fence most of the year but opened during the periods of nutritional constraints (i.e., end of the winter and summer). Other cervid species were present in the area (i.e., fallow deer *Dama dama* and roe deer *Capreolus capreolus*), but their densities were negligible.

From 2000 to 2011, the main management goals were to substantially reduce deer density (from over 40 to below 30 deer/km^2^) and to even sex ratio (1:1) to correct the existing female‐biased sex ratio (0.59 males per females). Therefore, a high percentage of the deer population was harvested each year (~20%), placing more emphasis on culling females than males (25% more females than males).

### 2.2 Data collection

We analyzed data on 4176 known‐age females culled (or found dead) mostly from October to February during the years 2000–2019. These females were a random subsample of the population (i.e., no hunting selection was performed because the objective was culling as much females as possible).

For each individual culled, the first incisor (I_1_) was removed to assess its age, although for younger ones, still with milk incisors, the age was assessed by tooth replacement and succession method (Azorit et al., 2002; Mitchell, 1967). Histological examinations of incisors were performed by staff at the Los Quintos de Mora's laboratory by counting cementum annuli (Azorit et al., 2002; Low & Cowan, 1963).

After extraction of the incisors, each tooth was placed into labeled plastic containers filled with water. They were left in the plastic containers for around four weeks to let the soft tissue rot away and thus help eliminating any remaining soft tissues before decalcification. Subsequently, incisors were placed in a 5% nitric acid solution for two or three days (Morris, 1972). Once decalcified, each tooth root was placed in a cryostat where several longitudinal cuts of 25 μm were made at a temperature of −18°C. The different sections were stained using diluted hematoxylin, and then, several sections of each tooth were mounted on a glass microscope slide. Finally, the dental cementum annuli were count by using a 20× magnification microscope (ZEISS MC 100).

### 2.3 Data analysis

#### 2.3.1 Age pyramid calculation

To construct the life table or age pyramids, cumulative data on mortality over the period 2000–2019 were used. It is important that this information extends over many generations after the last year for which we calculated the age pyramid as red deer are long‐lived species (life span = 15–18 years; Nussey et al., 2006; Loison et al., 1999). Therefore, we used data of known‐age females culled or found dead till year 2019 (*n* = 4176) to calculate age pyramid for the period 2000 to 2011. Furthermore, as the study area was fenced, there were no immigrant or emigrant individuals in our population which provided a perfect experimental ground to perform this type of analysis. Finally, as we knew the age of each female at death, we could calculate its age during the previous years. Therefore, if a given year (*t*) a 5‐year‐old hind was culled, we knew that during year (*t*−1), the female was 4 years old; during year (*t*−2), it was 3 years old; and so on.

As red deer are born in spring (April–June), each cohort comprised the period between the month of May of the year (*t*) until April of year (*t* + 1). However, note that almost 98% of the data was collected between September and February, the hunting season.

#### 2.3.2 Population parameters

Information about population size and sex ratio was very important to assess the reliability of the age pyramids as it is key to assess the percentage of the estimated female population for which we had age information. The higher this percentage, the higher the accuracy of the age pyramids. In addition, any change through time of both variables could have important implications for population demography (Bonenfant et al., 2009). Therefore, before drawing any conclusion, we confirmed that density and sex ratio variation was small between those years with severe drought events in order to minimize the possibility that any of these variables influenced the observed response.

##### Population size calculation

Yearly line transects were performed to assess population size in the estate. The survey comprised nine parallel transects (E‐W orientation) systematically spaced at least 1 km from each other to avoid double counting of individuals. Double counting during transects could occur only when deer run in the same direction of the observer movement. This is not usually the case as they run more often perpendicular to the direction of the observer but can sometimes happen. Therefore, we expect it not to have a great impact on the results. The total area surveyed was 21.47 km^2^, and the sampling effort was 7.94 km/10 km^2^. Surveys were performed during the rut (end of September–beginning of October) and were performed twice during consecutive days, firstly E‐W direction and secondly, W‐E. Almost every year, each transect was performed by the same observer. The information collected during the survey was the following: perpendicular distance to the transect line of the individual or group found, number of individuals in the group, sex, age class (fawns, yearlings and adults), and habitat type.

For the analysis, a post‐stratification method was used to estimate the specific detection function for each habitat type that combined main vegetation types and topographic features (i.e., three habitats: croplands and dehesas, shrublands, pine woodlands of the lowlands and two topographic features: lowlands and hillslopes, a total of 6 different habitats). Then, we fitted three detection functions (half‐normal cosine, uniform cosine, and hazard‐rate with simple polynomial adjustment) to each habitat stratum and selected the best model based on the lowest Akaike information criterion (AIC). We also tested correlations between cluster size and distance from the transect line searching for cluster size bias. When detected (often in croplands and dehesas), regression techniques were used to determine an unbiased cluster size estimate for density calculations (Buckland et al., 2004). Finally, density estimates and confidence intervals were computed according to distance sampling methodology (Buckland et al., 2004) using R software package “Distance” (R Development Core Team, 2018).

##### Sex ratio calculation

Sex ratio was calculated using information on both, known‐age males and females for each year. In addition to the female data, a total of 3725 known‐age males culled or found dead between 2000 and 2019 were used (see Peláez et al., 2018 for male information). At the beginning of the study, sex ratio was biased toward females as the hunting technique during the previous decade was focused on trophy hunting. However, as exposed above, from 2000 to 2010, one of the main management goals was to revert this situation by evening sex ratio.

#### 2.3.3 Environmental variables

##### Spring real bioclimatic index (spring RBI)

This index is an adaptation of Gaussen aridity index (Bagnouls & Gaussen, 1953) developed by Montero de Burgos and González‐Rebollar (1974) that measures monthly soil water availability for plant as an index of plant productivity. It uses meteorological information on monthly precipitation and average monthly temperature but also water runoff (we chose 10%, appropriate for soils on moderate slopes) and soil water retention capacity (100 mm, typical of most soils). It has been used previously in the study area (Martínez‐Jauregui et al., 2009; Peláez et al., 2018; Pelaez et al., 2017) to determine food availability for hinds during late gestation (March and April) and early lactation (May and June). Low mean values of RBI indicate low plant productivity as a result of drought (low precipitation and hot temperature) or winter vegetative rest (low temperatures). Spring plant productivity (from March to June) is very important in Mediterranean environments as a long productive spring shortens the deleterious effect of the summer drought, when females are lactating, allowing females to come into the rutting season with higher body reserves (Bugalho & Milne, 2003; San Miguel et al., 1999). Meteorological data were obtained from a weather station located at Los Cortijos, 2 km south of the estate and from Los Quintos de Mora weather station (Spanish National Meteorological Agency).

In order to identify extreme drought events, we firstly obtained historical meteorological data from a 40‐year period (from 1975 to 2015) and calculated, for each year, the value of spring RBI. Secondly, to stablish a selection criterion, we defined what we would consider an extreme drought event. We used the widely accepted definition proposed by the International Panel for Climate Change (IPCC, 2007): “An extreme weather event is rare at a particular place and time of year. Definitions of rare vary, but an extreme weather event would normally be as rare as or rarer than the 10th or 90th percentile of a probability density function estimated from observations.” Therefore, following this definition, we ranked all values of spring RBI during a period of 40 years and selected those years being at the 10th percentile (i.e., lowest RBI and thus, the years of extreme drought). These years were, from low to high, 2005, 1995, 2003, and 1990, although only two of these extreme drought events occurred during our study period (2005 and 2003).

## 3 RESULTS

For our population, we obtained a yearly female age structure based on a mean of 953 ± 120 (± *SD*) known‐age females which corresponded to more than 80% ± 12 (mean ± *SD*) of the total estimated female population (*N*_f_ = 1219 ± 254 females per year; mean ± *SD*; see Table 1). This high percentage meant that most of the females living in the estate, sooner or later, ended up being hunted as a consequence of the intense culling pressure in the estate (22% ± 9; mean ± *SD* of female harvested each year).

**TABLE 1:** Percentage of the total estimated female population used to construct the retrospective life table

Year	Estimated population size (*N*)^a^	Known‐age Individuals		Sex ratio^b^ (males:female)	Est. female population (*N*_f_)	% of total females used in pyramids	Harvested hinds/year	% Harvested females
		Males	Females					
2000	2467	662	1114	0.59	1547	72	436	28
2001	2773	677	882	0.77	1569	56	139	9
2002	2516	820	1020	0.8	1395	73	267	19
2003	2666	869	1037	0.84	1450	71	269	19
2004	2564	872	1005	0.87	1373	73	221	16
2005	2345	871	1016	0.86	1263	80	259	21
2006	2127	763	919	0.83	1162	79	128	11
2007	1924	902	1024	0.88	1023	100	226	22
2008	2021	891	994	0.9	1066	93	236	22
2009	2071	896	962	0.93	1072	90	347	32
2010	1995	887	781	1.14	934	84	247	26
2011	1832	994	683	1.38	769	89	298	39
Mean ± *SD*	2275 ± 318	838 ± 90	953 ± 120	0.9 ± 0.2	1219 ± 254	80 ± 12	256 ± 83	22 ± 9

^a^ Estimated population size was obtained from survey data.

^b^ Sex ratio was obtained from individuals known to be alive based on female and male data.

Furthermore, by combining information on the number of known‐age individuals (males and females) each year, we were able to calculate sex ratio during the study period (Table 1). Thus, we could observe an adjustment of the sex ratio from being female‐biased at the beginning of the study (0.6 males per female) to achieve a more even structure (1.1 males per female in 2010) or even male‐biased sex ratio at the end (1.4 males per female in 2011). Mean deer population density decreased throughout the years (41.9 deer/km^2^ in 2001 and 29.6 deer/km^2^ in 2011; See Table S1).

**FIGURE 1:**
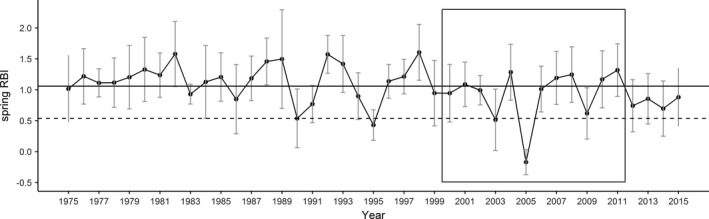
Mean Real Bioclimatic Index of spring (March, April, May, and June) from a period of 40 years (1975–2015). Error bars indicate standard deviation (*SE*). Solid horizontal line indicates mean spring RBI for the whole period. Dashed horizontal line represents the 10th percentile of lower values of RBI. The years for which we calculated the age pyramids are inside the black square

In addition, during the study period (2000–2011), we could observe two years (2003 and 2005) that could be classify as extreme events (i.e., being at the 10th percentile of lowest spring RBI index for a 40‐year period; Figure 1). Year 2005 was the least favorable year for resource availability, with the lowest RBI index. In addition, population density was high in 2003 and 2005 (40 and 36 ind/100 ha, respectively) and sex ratio variation was modest (0.84 and 0.86 males per female, respectively).

### 3.1 Effect of drought on demography

**FIGURE 2:**
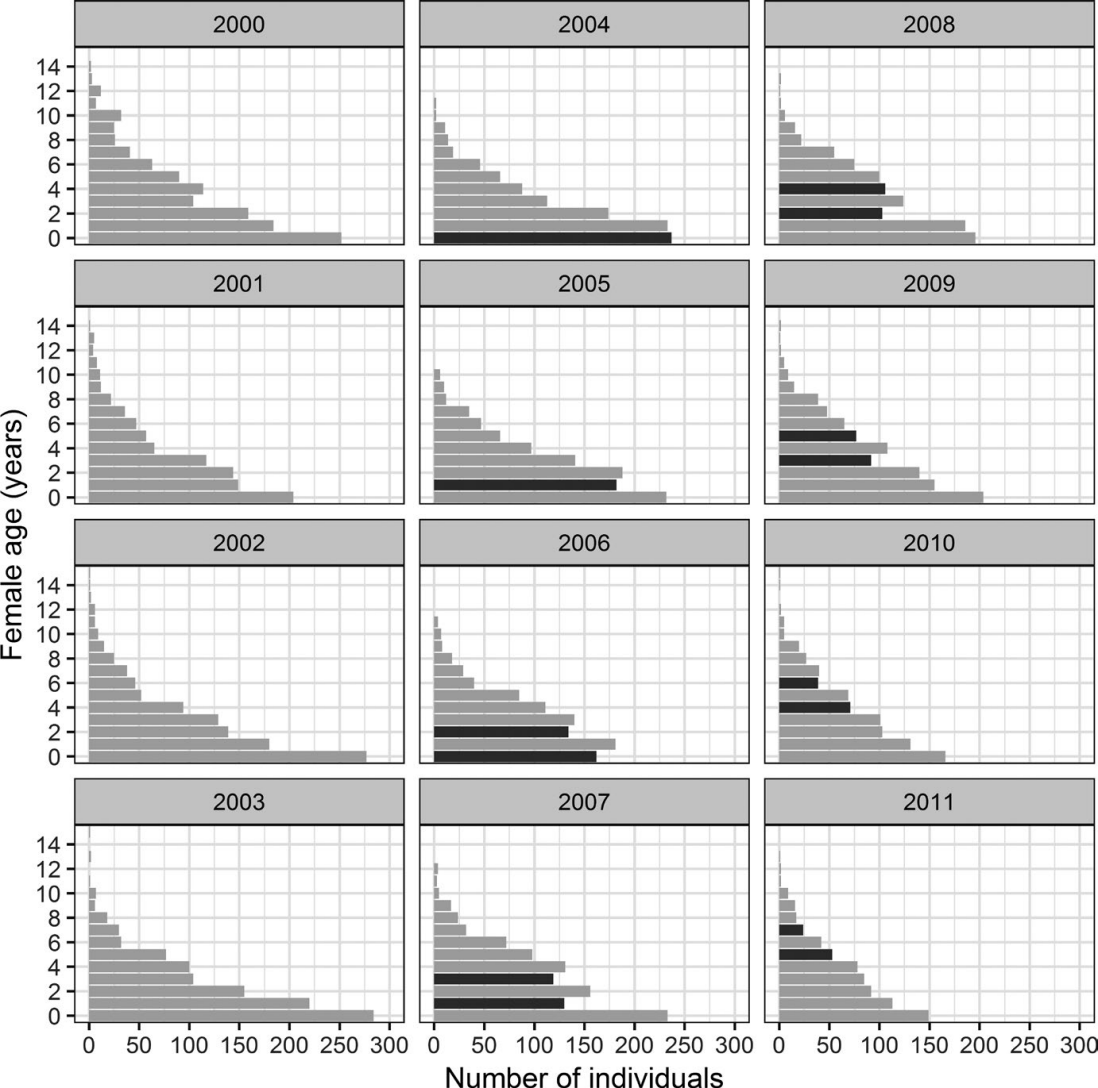
Female age pyramids during the study period (2000–2011). Highlighted in dark gray, the cohorts 2004 and 2006 that were born after the drought years with higher density (2003 and 2005, respectively)

Averaged age structure of the female population indicated that 23% of them were fawns, 18% yearlings, 15% two years old, and 12% three years old (see Table S2). This decrease in the number of individuals as the age‐group increases represents the normal shape of an age pyramid (i.e., the size of the younger cohorts is bigger than the size of the older ones). However, after dry‐spring years with higher density (2003 and 2005), we could see some major deviations from the normal age structure of the population. In 2004, after 2003 drought, the fawn age‐group represented almost the same percentage of the female population than the yearling group (24% vs. 23%; See Figure 2, Video 1 and Table S2). Similarly, in 2006, after the extreme drought event of 2005, the fawn cohort was even smaller than the yearling group (18% fawns vs. 20% yearlings), reversing the normal decreasing trend in the percentage of individuals with increasing age‐group. Furthermore, during the following 3 years, both cohorts (born in 2004 and 2006) maintained this reverse relationship, representing a smaller percentage of the population than the previous' year cohort.

**VIDEO 1:** Female age‐structure variation during the study period (2000–2011). Highlighted in red, cohorts born in 2004 and 2006 after two drought years with high density (2003 and 2005, respectively)

## 4 DISCUSSION

By using retrospective life tables, we demonstrated the strong demographic effects that extreme dry events can have on red deer population dynamics in Mediterranean environments, presumably exacerbated by the high population density conditions. Furthermore, we observed lasting cohort effects in our population produced by several drought events that, to the best of our knowledge, have not been reported before with such clarity for any other Northern Hemisphere cervid population [but see Gaillard et al. (2013) on roe deer].

Overall, retrospective life tables seem to reflect the effect of extreme weather events on red deer population demography in Mediterranean environments as, indeed, the demographic changes represented by the age pyramids were in accordance with previous findings from the same population and period of time (Peláez et al., 2018; Pelaez et al., 2017; Rodríguez‐Hidalgo et al., 2010). Two main reasons may have contributed to the reduction of the fawn cohort observed after those dry events (2003 and 2005): (a) a decrease in pregnancy rates and (b) later conception dates and thus delayed parturition dates. Indeed, in the study area, pregnancy rates between October–February were below average after both dry years [see season 2003–2004 and 2005–2006 in Figure 1C from Rodríguez‐Hidalgo et al. (2010)], which may have produced an overall reduction in the number of fawns born the following year. In addition, the rest of females that did get pregnant conceived later as they may have had to wait for the autumnal rains or the acorn crop to recover body condition to be able to ovulate (Pelaez et al., 2017). Certainly, in the study area, following 2003 and 2005 drought events, there was a delay in conception dates at population level (an average of 6 and 10 days, respectively; Pelaez et al., 2017), and thus, presumably, fawns were born later. Delayed parturition dates may cause a mismatch between resource availability and energetic needs of hinds and fawns, increasing stillbirths, newborn‐fawn mortality, and over‐summer calf mortality (Clutton‐Brock, Iason, et al., 1987; Clutton‐Brock, Major, et al., 1987; Plard et al., 2014). In Mediterranean environments, milk quality and yield were significantly reduced when comparing late‐calving hinds to those that gave birth early in the season (Landete‐Castillejos et al., 2000). Thus, those females that give birth later in the season experience food restriction as resource availability sharply decreases during the summer drought which may have reduced calf growth and, ultimately, increased fawn mortality (Landete‐Castillejos et al., 2003).

Interestingly, our results show lasting effects on the population structure, at least for 3 years after the extreme drought events occurred. This is the first time a study showed this lasting effect with such clarity for any Northern Hemisphere cervid population by using age pyramids. Lowe (1969), who studied red deer living in Rhum Island (Scotland) for 5 years, was the only study found reporting retrospective age pyramids for the species but no single year showed such disruption in the age pyramids as the one observed in our population. Unfortunately, the lack of studies reporting time‐specific life tables for red deer obtained from empirical data prevented us from comparing our results with that from other populations. From the literature, we could, however, retrieve information from other cervid populations inhabiting artic or alpine environments that also reported strong cohort effects and population crashes as a consequence of the interaction between high‐density conditions and above‐average snowfalls during winter [see Peterson (1999) on moose *Alces alces* L.; Caughley & Gunn (1993) on caribou *Rangifer tarandus* L.; Garrott et al. (2003) on elk *Cervus elaphus*]. Indeed, both climatic extremes (i.e., drought in Mediterranean environments and heavy snowfalls in artic/alpine areas) can potentially reduce the supply of available forage (Caughley & Gunn, 1993) and thus drastically reduce fawn recruitment (Peterson, 1999) or even increase overall population mortality (Caughley & Gunn, 1993). All three regions constitute the edge of the species range of distribution, and therefore, they impose a severe environmental constraint on the life histories of these cervid species (Stearns, 1992). However, one of the main differences between these environments is the distribution of resources throughout the year. While artic and alpine habitats display a high intra‐annual variation in food availability producing high fawn and adult mortality rates during winter (Bonardi et al., 2017), Mediterranean environments provide a permanent source of low–medium food quality for deer (i.e., evergreen shrubs and trees) even during the periods of nutritional constraint (i.e., summer; San Miguel et al., 1999), which may be less likely to produce adult mortality (see Gaillard et al., 2013 on roe deer). As a consequence, the number of deer that Mediterranean environments can support is usually higher compared with other temperate or artic regions (Acevedo et al., 2008). We believe that our results may have been exacerbated by the combined negative effects that population parameters (such as density) and extreme drought events can exert on the population dynamics, although the lack of contrasting data on years with high and low density prevented us from drawing any conclusions.

As predicted by the IPCC, drought events will become more frequent and intense under the current climate change context (IPCC, 2013). This, together with the increasingly higher ungulate populations in most areas of the Mediterranean Basin, mostly due to land‐use changes and current game management practices (Carpio et al., 2017), might cause unforeseen effects on population dynamics. However, it is also true that extreme climate events may help control the overabundant populations by increasing the costs of reproduction and thus reducing reproductive success. But, before this happen, there might be undesired effects on other species and on ecosystem integrity such as the negative impacts of deer browsing on vegetation composition and structure (Mysterud, 2006; Perea et al., 2014) or the appearance of zoonotic diseases (Gortázar et al., 2015), among others. Therefore, it seems more sensible to maintain low deer densities in Mediterranean environments to help red deer counteract the expected negative effects of climate change.

Most of the studies using retrospective data focused on calculation of population size (Fryxell et al., 1988; Lowe, 1969; McCulloug, 1979). However, the use of this methodology to calculate density is very controversial as it tends to underestimate population size (Roseberry & Woolf, 1991). Other studies used the data to calculate average population age structure throughout a period of time instead of yearly age structure (Fruziński & Łabudzki, 1982 using roe deer hunting data; Rehnus et al., 2018 on marked and recaptured roe deer fawns). Although the use of retrospective data in this study may not be useful to extract population dynamic parameters such as mortality rates as data are collected from heavily managed populations, and thus, most of the individuals should end up being hunted at some point of their lives, it could provide useful lessons to: (a) improve our understanding of the inter‐annual variation in recruitment associated with climate change (extreme weather events, trends on global climate, etc.) and (b) assess retrospectively the effects of culling/management plans. Indeed, for the latter, it is clear that the higher culling pressure exerted over females in our population helped to achieve both main management goals, to even sex ratio and also to reduce population density (see also Pelaez et al., 2017). As hunting data are nowadays largely available throughout the Northern Hemisphere and many sites also accurately record the age of individuals, we would like to highlight the value of this methodology to potentially address the effect of climate change on ungulate population demography.

## CONFLICT OF INTEREST

No potential competing interest was reported by the authors.

## AUTHOR CONTRIBUTION

**Marta Peláez:** Formal analysis (lead); Methodology (lead); Software (lead); Visualization (lead); Writing‐original draft (lead); Writing‐review & editing (lead). **Alfonso San Miguel:** Conceptualization (equal); Supervision (supporting). **Carlos Rodriguez‐Vigal:** Conceptualization (equal); Funding acquisition (equal); Project administration (lead); Supervision (supporting). **Ángel Moreno‐Gómez:** Project administration (supporting); Supervision (supporting). **Amanda García del Rincón:** Data curation (lead). **Ramon Perea:** Funding acquisition (equal); Supervision (lead); Writing‐review & editing (supporting).

## Supporting information


**Table S1.** Results of population estimation using distance sampling methodology. Total area used to calculate density was 66.12 km2.
**Table S2.** Female age structure during the study period (2000‐2010). Red years indicate a pyramid age disruption, with an increase in the percentage of a cohort with increasing age group.
**Figure S1.** Culled females by year and age.
**Figure S2.** Composition of pyramids according to the year when each female was culled or found dead.

## Data Availability

Data associated with this manuscript are deposited in the Dryad Digital Repository: https://doi.org/10.5061/dryad.fn2z34tvd.
