# Supplementary material for: Using retrospective life tables to assess the effect of extreme climatic conditions on ungulate demography
Source: Ecol Evol. 2021 Dec 24;12(1):e8218. doi: 10.1002/ece3.8218 (PMC8794714; doi:10.1002/ece3.8218)

**Supporting information**

**Table S1.** Results of population estimation using distance sampling methodology. Total area used to calculate density was 66.12 km2.

|  | Estimated  population size (N) | | Estimated population density  (D=ind/km^2^) | |  |
| --- | --- | --- | --- | --- | --- |
| Year | **N total** | **[LCL, UCL]** | **D total** | **[LCL, ULC]** | |
| 2000 | 2467 | [1586, 3788] | 37.32 | [23.99, 57.29] | |
| 2001 | 2773 | [1566, 4583] | 41.94 | [23.68, 69.31] | |
| 2002 | 2516 | [1646, 3820] | 38.05 | [24.90, 57.78] | |
| 2003 | 2666 | [1315, 4692] | 40.32 | [19.88, 70.97] | |
| 2004 | 2564 | [1645, 3943] | 38.79 | [24.88, 59.63] | |
| 2006 | 2127 | [1429, 3175] | 32.18 | [21.61, 48.02] | |
| 2007 | 1924 | [1201, 3009] | 31.04 | [18.17, 45.51] | |
| 2008 | 2021 | [1400, 2951] | 32.59 | [21.18, 44.63] | |
| 2009 | 2071 | [1242, 3315] | 33.41 | [18.79, 50.15] | |
| 2010 | 1995 | [1409, 2873] | 32.18 | [21.32, 43.46] | |
| 2011 | 1832 | [1279, 2661] | 29.55 | [19.35, 40.25] | |
| Average | 2269 | [1429, 3528] | 35.21 | [21.61, 53.36] | |

**Table S2.** Female age structure during the study period (2000-2010). Red years indicate a pyramid age disruption, with an increase in the percentage of a cohort with increasing age group.

| Female age | Year | | | | | | | | | | |  |  | |
| --- | --- | --- | --- | --- | --- | --- | --- | --- | --- | --- | --- | --- | --- | --- |
|  | **2000** | **2001** | **2002** | **2003** | **2004** | **2005** | **2006** | **2007** | **2008** | **2009** | **2010** | **2011** | **Mean** |  |
| 0 | 0.23 | 0.23 | 0.27 | 0.27 | **0.24** | 0.23 | **0.18** | 0.23 | 0.2 | 0.21 | 0.21 | 0.22 | **0.23** |  |
| 1 | 0.17 | 0.17 | 0.18 | 0.21 | 0.23 | **0.18** | 0.20 | **0.13** | 0.19 | 0.16 | 0.17 | 0.17 | **0.18** |  |
| 2 | 0.14 | 0.16 | 0.14 | 0.15 | 0.17 | 0.19 | **0.15** | 0.15 | **0.10** | 0.15 | 0.13 | 0.13 | **0.15** |  |
| 3 | 0.09 | 0.13 | 0.13 | 0.10 | 0.11 | 0.14 | 0.15 | **0.12** | 0.12 | **0.10** | 0.13 | 0.12 | **0.12** |  |
| 4 | 0.10 | 0.07 | 0.09 | 0.10 | 0.09 | 0.10 | 0.12 | 0.13 | 0.11 | 0.11 | 0.09 | 0.11 | **0.10** |  |
| 5 | 0.08 | 0.06 | 0.05 | 0.07 | 0.07 | 0.06 | 0.09 | 0.10 | 0.10 | 0.08 | 0.09 | 0.08 | **0.08** |  |
| 6 | 0.06 | 0.05 | 0.05 | 0.03 | 0.05 | 0.05 | 0.04 | 0.07 | 0.08 | 0.07 | 0.05 | 0.06 | **0.05** |  |
| 7 | 0.04 | 0.04 | 0.04 | 0.03 | 0.02 | 0.03 | 0.03 | 0.03 | 0.06 | 0.05 | 0.05 | 0.04 | **0.04** |  |
| 8 | 0.02 | 0.02 | 0.02 | 0.02 | 0.01 | 0.01 | 0.02 | 0.02 | 0.02 | 0.04 | 0.03 | 0.02 | **0.02** |  |
| 9 | 0.02 | 0.01 | 0.01 | 0.01 | 0.01 | 0.01 | 0.01 | 0.02 | 0.02 | 0.02 | 0.03 | 0.02 | **0.01** |  |
| 10 | 0.03 | 0.01 | 0.01 | 0.01 | <0.01 | 0.01 | 0.01 | <0.01 | 0.01 | 0.01 | 0.01 | 0.01 | **0.01** |  |
| 11 | 0.01 | 0.01 | 0.01 | <0.01 | <0.01 | 0 | <0.01 | <0.01 | <0.01 | 0.01 | 0.01 | <0.01 | **<0.01** |  |
| 12 | 0.01 | <0.01 | 0.01 | 0 | 0 | 0 | 0 | <0.01 | <0.01 | <0.01 | <0.01 | <0.01 | **<0.01** |  |
| 13 | <0.01 | 0.01 | <0.01 | 0 | 0 | 0 | 0 | 0 | <0.01 | <0.01 | 0 | <0.01 | **<0.01** |  |
| 14 | <0.01 | <0.01 | 0 | 0 | 0 | 0 | 0 | 0 | 0 | <0.01 | <0.01 | 0 | **<0.01** |  |
| 15 | 0 | 0 | <0.01 | 0 | 0 | 0 | 0 | 0 | 0 | 0 | <0.01 | 0 | **<0.01** |  |

**Figure S1.** Culled females by year and age.


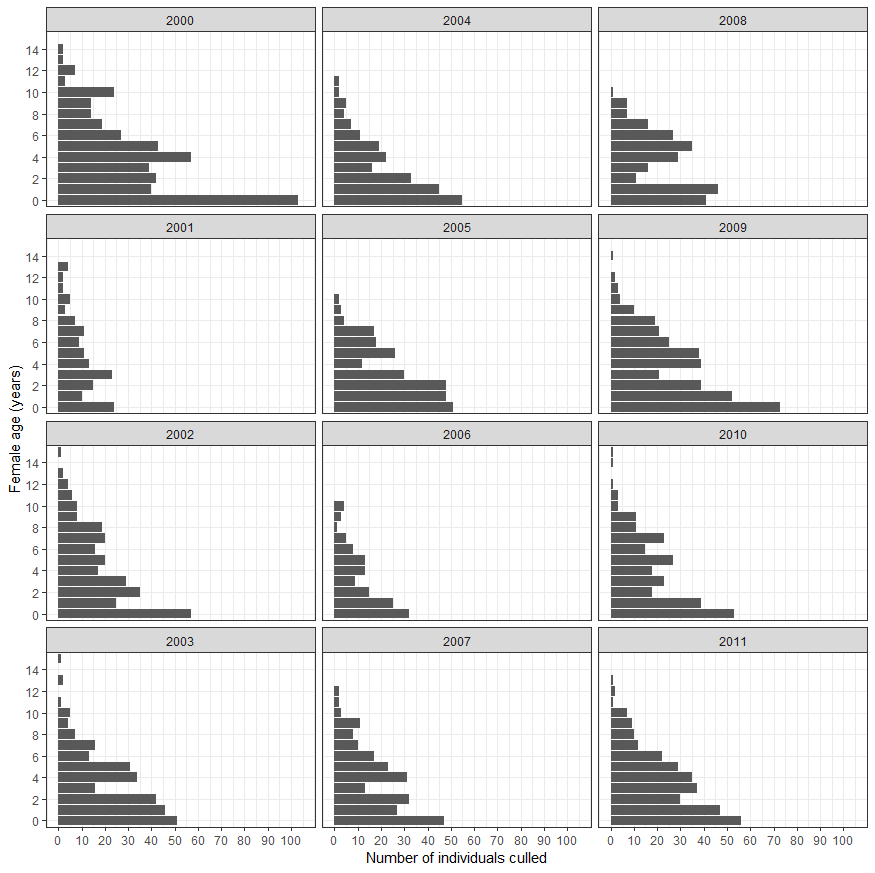


**Figure S2.** Composition of pyramids according to the year when each female was culled or found dead.


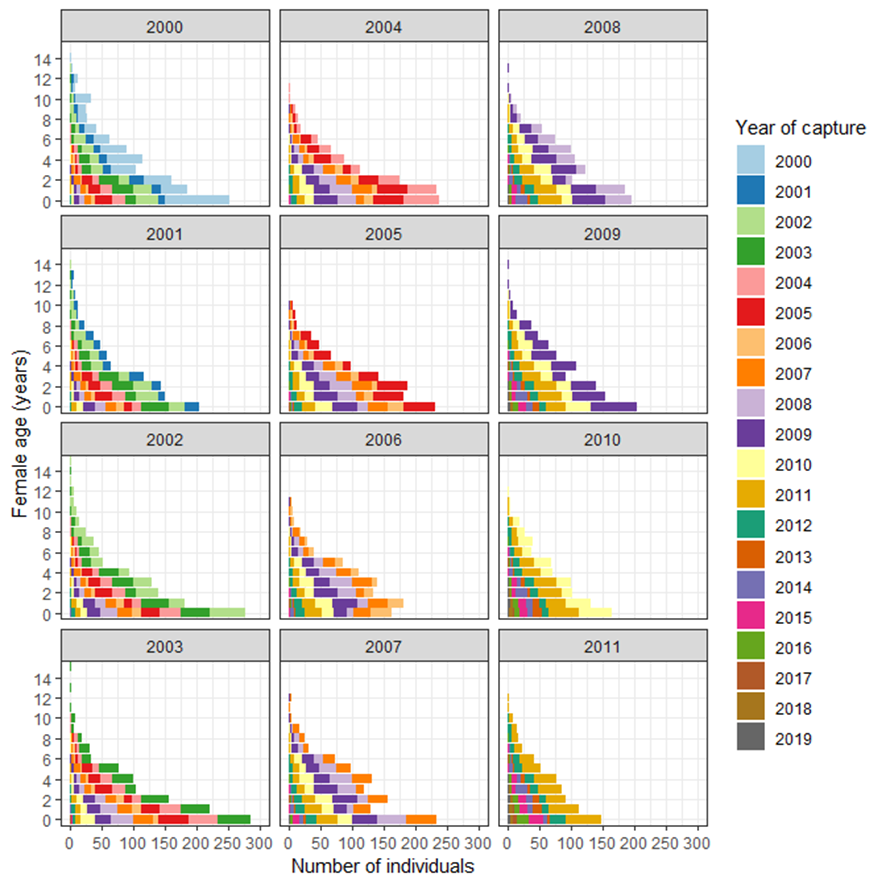

Supplement: Supplementary file 1 — Table S1. Results of population estimation using distance sampling methodology. Total area used to calculate density was 66.12 km2. Table S2. Female age structure during the study period (2000‐2010). Red years indicate a pyramid age disruption, with an increase in the percentage of a cohort with increasing age group. Figure S1. Culled females by year and age. Figure S2. Composition of pyramids according to the year when each female was culled or found dead. [file ECE3-12-e8218-s001.docx]
